# Single Assessment Numeric Evaluation (SANE) – ein vielversprechendes, valides Messinstrument der „patient-reported outcome measures“ (PROM)

**DOI:** 10.1007/s00132-021-04190-w

**Published:** 2021-11-11

**Authors:** Marcus Örgel, Tilman Graulich, Emmanouil Liodakis

**Affiliations:** Klinik für Unfallchirurgie, Medizinische Hochschule Hannover, Carl-Neuberg-Straße 1, 30625 Hannover, Deutschland

**Keywords:** Oxford Elbow Score, Patient-reported outcome measures, Single Assessment Numeric Evaluation, Visuelle Analogskala, Oxford Elbow Score, Patient-reported outcome measures, Single Assessment Numeric Evaluation, Visual analog scale

## Abstract

„Patient-reported outcome measures“ (PROM) sind klinische Messinstrumente, die den Gesundheitszustand sowie die Ergebnisse medizinischer Interventionen aus der Perspektive des Patienten erfassen. Ziel dieser Studie war es, retrospektiv die Korrelation zwischen dem Oxford Elbow Score (OES) und Single Assessment Numeric Evaluation (SANE) zu untersuchen. Dies wurde zwischen Dezember 2018 und Februar 2019 bei 86 Patienten mittels des Pearson Korrelationskoeffizient zwischem dem SANE und OES untersucht. OES und die SANE korrelierten signifikant (r = 0,903, *p* < 0,001), sodass SANE als vielversprechender Outcome-Parameter neben etablierten Scores betrachtet werden kann.

## Abstract (en)

Patient-reported outcome measures (PROMs) are clinical measurement tools that capture the health status and outcomes of medical interventions from the patient’s perspective. The aim of this study was to investigate the correlation between the Oxford Elbow Score (OES) and Single Assessment Numeric Evaluation (SANE) retrospectively. The study examined 86 patients between December 2018 and February 2019 using the Pearson correlation coefficient between SANE and OES. OES and SANE correlated significantly (*r* = 0.903, *p* < 0.001), so that SANE can be considered a promising outcome parameter alongside established scores.

## Originalpublikation

Razaeian S et al (2020) Correlation between Oxford Elbow Score and Single Assessment Numeric Evaluation: Is one simple question enough? J Shoulder Elbow Surg 29(6):1223–1229. 10.1016/j.jse.2020.01.067

## Hintergrund

„Patient-reported outcome measures“ (PROM) sind klinische Messinstrumente, die den Gesundheitszustand sowie die Ergebnisse medizinischer Interventionen aus der Perspektive des Patienten erfassen. Sie nehmen in der Medizin eine immer bedeutendere Stellung ein. In der Ellenbogenchirurgie ist derzeit der methodisch am besten validierteste PROM der Oxford Elbow Score (OES). Ziel dieser Studie war es, retrospektiv die Korrelation zwischen dem OES und der bisher noch am Ellenbogen ungeläufigen Single Assessment Numeric Evaluation (SANE) zu untersuchen. Hierbei handelt es sich um eine subjektive Bewertung des aktuellen Gesundheitszustandes zwischen 0 und 100 % durch den Patienten vor dem Hintergrund, dass ein in diesem Falle vollständig gesundes und funktionsfähiges Ellenbogengelenk 100 Punkte hätte. Dies wird in Form einer einzigen Frage formuliert. O’Connor et al. [1] publizierten in ihren Arbeiten vergleichbare Ergebnisse zu PROM. Zu einem der weltweit am häufigsten genutzten PROM zählt die Visuelle Analogskala [2].

## Methode

Zwischen Dezember 2018 und Februar 2019 wurde in 107 Konsultationen bei 86 Patienten der OES und die SANE erhoben. Es wurde der Pearson-Korrelationskoeffizient zwischen dem OES, inkl. seiner drei Subdomänen – Schmerz, Funktion, Psychosozialität – und der SANE ermittelt. Durch eine lineare Regressionsanalyse sollten mögliche Prädiktoren (Alter, Geschlecht, Diagnose, Grund der Vorstellung, Schmerzniveau) für eine stärkere Korrelation identifiziert werden.

## Ergebnisse

Der OES und die SANE korrelierten signifikant (r = 0,903, *p* < 0,001). Unter Betrachtung der einzelnen Subdomänen korrelierte in absteigender Reihenfolge der psychosoziale Faktor (r = 0,885, *p* < 0,001) gefolgte von OES-Funktion (r = 0,847, *p* < 0,001) und OES-Schmerz (r = 0,804, *p* < 0,001) am stärksten mit der SANE. Eine inverse Korrelation zeigte sich zwischen der Visuellen Analogskala (VAS) und der SANE (r = −0,631). Zudem konnte die VAS als unabhängiger Prädiktor für den OES identifiziert werden.

## Schlussfolgerung

Die SANE korreliert hochsignifikant mit dem OES und ist ein einfach zu erhebender Funktionsscore, der weder gebühren- noch lizensierungspflichtig ist und als vielversprechender Outcome-Parameter neben etablierten Scores betrachtet werden kann.

## Kommentar

### Studiendesign

Das partielle Review [3] erfolgte unter Berücksichtigung der Standards der STARD-Initiative. Diese retrospektive Kohortenanalyse untersucht transparent den Zusammenhang zwischen zwei Funktionsscores (OES versus SANE). Hierbei erläutern die Autoren detailliert ihre Methodik. Sie gehen ausführlich auf die Rekrutierung der Studienpopulation ein. Als Referenzstandard wurde der etablierte Oxford Elbow Score [4] verwendet, welcher von der Oxford University Innovation (vormals bekannt als Isis Innovation Ltd., Oxford, UK) entwickelt wurde und lizenz- sowie gebührenpflichtig ist. Er besteht aus insgesamt 12 Fragen und ist derzeit der methodisch am besten validierte Funktionscore für das Ellenbogengelenk [5, 6].

Für die statistische Auswertung werden der Pearson-Korrelationskoeffizient und eine lineare multivariante Regressionsanalyse verwendet. Zudem wurde zur Quantifizierung der Unsicherheit das Konfidenzintervall (CI 95 %, *p* < 0,005) angeben. Wünschenswert wäre eine genaue Darlegung des Umgangs mit der Verzerrung von Daten gewesen.

### Interpretation der Ergebnisse

Die signifikanten Ergebnisse sind nachvollziehbar und haben eine hohe statistische Aussagekraft. Allerdings muss berücksichtigt werden, dass 14 Patienten mehrfach evaluiert wurden. Dies entspricht 16,3 % der Gesamtkohorte. Ob dies statistisch relevant gewesen wäre bzw. ob dies in der statistischen Auswertung berücksichtigt wurde, kann an dieser Stelle nicht beantwortet werden. Jedoch wurden hieraus zwei Gruppen [„pre-treatment“ versus „post-treatment“] verglichen, die nach ihrer statistischen Auswertung, die o. g. signifikanten Ergebnisse bestätigten, von der Gruppengröße jedoch äußerst klein waren. Ferner waren die Nachuntersuchungsintervalle sowie die eingeschlossenen Behandlungsprozeduren in dieser Subgruppenanalyse sehr heterogen.

Des Weiteren ist kritisch zu bedenken, dass die Scores jeweils nur ein einziges Mal und nur von einem einzigen Observer erhoben worden sind. Eine Inter- oder Intraobserver-Reliabilitätsanalyse fehlt ebenso wie eine Test-Retest-Reliabilitätsanalyse.

### SANE ein weiterer effektiver PROM für den klinischen Alltag

Dennoch belegen die Ergebnisse eine stark signifikante Korrelation und stellen SANE einmal mehr als vielversprechenden PROM dar, der bereits in der Schulter- und Kniechirurgie ähnliche Eigenschaften unter Beweis stellen und dort neben etablierten Funktionsscores einen vergleichbaren Stellenwert einnehmen konnte [7].

Nicht von ungefähr hat bereits vor einigen Jahre das sogenannte Value Commitee der American Shoulder and Elbow Surgeons (ASES) mit Blick auf über 25 verschiedene PROM auf dem Gebiet der Ellenbogen- und Schulterchirurgie SANE als einen von drei nennenswerten Baseline-Funktionsscores für den klinischen Alltag zur Evaluierung von Ellenbogenbeschwerden empfohlen.

## Fazit für die Praxis

- „Patient-reported outcome measures“ (PROM) wie die Single Assessment Numeric Evaluation (SANE) sollten im Klinikalltag zur Evaluierung von Beschwerden eingesetzt werden.
- SANE ist ein vielversprechender, evidenzbasierter PROM auch zur Evaluierung von Ellenbogenbeschwerden.
- SANE ist weder lizensierungs- noch gebührenpflichtig.
- SANE kann mit einem geringen zeitlichen Aufwand angewendet werden und liefert verwertbare Aussagen hinsichtlich der Beschwerden des Ellenbogengelenkes.

## Abkürzungen

ASES: American Shoulder and Elbow Surgeons
CI: Konfidenzintervall
OES: Oxford Elbow Score
PROM: „Patient-reported outcome measures“
SANE: Single Assessment Numeric Evaluation
STARD: Standards for Reporting of Diagnostic Accuracy
VAS: Visuelle Analogskala
